# The Role of Aromatase Inhibitors in Male Prolactinoma

**DOI:** 10.3390/jcm12041437

**Published:** 2023-02-10

**Authors:** Amit Akirov, Yaron Rudman

**Affiliations:** 1 Institute of Endocrinology, Beilinson Hospital, Petach Tikva 49100, Israel; 2 Sackler School of Medicine, Tel Aviv University, Tel Aviv 6997801, Israel

**Keywords:** aromatase inhibitors, pituitary, prolactinoma

## Abstract

Background: dopamine agonists are the recommended treatment for male prolactinomas, but some patients may develop dopamine-agonist-resistant hyperprolactinemia, leading to persistent hypogonadism that requires treatment with testosterone. However, testosterone replacement therapy may be associated with a decrease in the efficacy of dopamine agonists due to the aromatization of testosterone to estradiol, which can stimulate the proliferation and hyperplasia of lactotroph cells in the pituitary, inducing resistance to dopamine agonists. Objective: this paper systematically reviewed the role of aromatase inhibitors for men with prolactinoma and dopamine-agonist-resistant or persistent hypogonadism following treatment. Method: we performed a systematic review of all studies (according to PRISMA guidelines), assessing the role of aromatase inhibitors, including anastrozole and letrozole, for male prolactinoma. An English-language search for relevant studies was conducted on PubMed from its inception to 1 December 2022. The reference lists of the relevant studies were also reviewed. Results: our systematic review identified six articles (nine patients), including five case reports and a single case series, on the use of aromatase inhibitors for male prolactinomas. Reducing estrogen levels with an aromatase inhibitor improved sensitivity to dopamine agonists, as the addition of anastrozole or letrozole improves the control of prolactin levels and may lead to the shrinkage of tumors. Conclusion: aromatase inhibitors are of potential value to patients with dopamine-agonist-resistant prolactinoma, or when hypogonadism persists while using high-dose dopamine agonists.

## 1. Introduction

Prolactin-producing pituitary adenoma, or prolactinoma, is in almost all cases a benign pituitary tumor that expresses and secretes prolactin, leading to hyperprolactinemia. Prolactinoma is the most common type of hormone-secreting pituitary tumor and accounts for about 40% of all pituitary adenomas. The prevalence of clinically apparent prolactinomas ranges from 6–10 per 100,000 to 50 per 100,000, according to different studies [1,2,3].

The symptoms of prolactinoma may be secondary to the tumor mass effect or secondary to hyperprolactinemia. In some cases, prolactinomas do not cause any noticeable signs or symptoms.

The tumor mass effect may be associated with neurological symptoms, headaches, visual disturbances, and symptoms secondary to the under-secretion of other pituitary hormones. A prolactinoma is classified based on its size, according to magnetic resonance imaging. The classes are as follows: a microprolactinoma, defined as a pituitary tumor <10 mm diameter, or a macroprolactinoma, defined as a pituitary tumor ≥10 mm diameter [4]. While prolactinomas are significantly more common in women than in men, the latter group harbors larger tumors. Studies have shown that prolactinomas in men are more invasive and show histologic evidence of more rapid growth; therefore, symptoms secondary to the tumor mass effect are more common among men than among women [1,5].

Symptoms secondary to high prolactin levels in premenopausal women may include hypogonadism and hypoestrogenism with infertility, galactorrhea, low libido, vaginal dryness, dyspareunia, and low bone density. Increased prolactin levels inhibit the pulsatile secretion of gonadotropin-releasing hormones by interfering with hypothalamic kisspeptin-secreting cells, resulting in menstrual cycle dysfunction, including oligomenorrhea or amenorrhea, and infertility [6]. As postmenopausal women are already hypogonadal, with low estrogen levels, they do not present with these classical symptoms and are usually diagnosed when large adenomas produce headaches or impaired vision, or as an incidental finding following imaging undertaken for another reason. However, under estrogen replacement therapy, hyperprolactinemic postmenopausal women may experience galactorrhea [7].

Hyperprolactinemia that is secondary to pituitary lactotroph tumors can also reduce testosterone levels in men and may result in impaired spermatogenesis, leading to infertility, erectile dysfunction, decreased libido, and low bone density. Low testosterone is also associated with anemia and decreased muscle mass. Galactorrhea may develop in men with hyperprolactinemia, though this is less common than in women, as male mammary tissue is less susceptible to the lactogenic effects of hyperprolactinemia [4,8].

The treatment goals for a prolactinoma include the normalization of prolactin levels and associated signs and symptoms, along with complete tumor removal or shrinkage and a reversal of the tumor mass effects. While transsphenoidal surgical resection is regarded as the first-line treatment for most secretory pituitary tumors, the recommended treatment for patients harboring prolactin-secreting pituitary adenomas involves dopamine agonists, aimed at reducing serum prolactin levels, decreasing tumor size, and restoring gonadal function [3,9].

Cabergoline is regarded as the first choice, while bromocriptine is usually reserved as a second-line treatment. Quinagolide is another dopamine agonist, which is not available in many countries [10].

Cabergoline, an ergot derivative with a high affinity for lactotroph dopamine 2 receptors, is a long-acting dopamine agonist, usually administered once or twice weekly. The starting dose is 0.25–0.5 mg/week and is increased until normal prolactin levels have been restored. Studies have shown normalization of prolactin levels in approximately 70–80% of patients; tumor size reduction was documented in about 60% of patients, with the resolution of galactorrhea, amenorrhea, infertility, and sexual function in more than half of patients [3,11].

Potential side effects associated with cabergoline treatment include nausea, which is reported in up to one third of patients, orthostatic hypotension or headache, documented in about a quarter of cases, and, rarely, dyskinesia. Additional side effects may include constipation, dyspepsia, fatigue, and rhinitis. Addictive and compulsive behaviors, such as gambling addiction, hypersexuality, food compulsions, or impulsive purchases, have been reported with cabergoline and are linked to the affinity of the medication for dopamine 3 receptors in the mesolimbic system. The risk for valvular heart disease associated with cabergoline is debated; however, at the lower doses generally used for prolactinomas, valvular disease is highly unlikely [12,13].

Bromocriptine, a semisynthetic ergot alkaloid dopamine agonist, is given twice daily and is considered less efficacious and tolerable than cabergoline. Prolactin normalization is reported in approximately 70–80% of patients, and a decrease in the pituitary tumor size of ~50% occurs in approximately 65% of patients. Bromocriptine is less well-tolerated compared with cabergoline, and, in these cases, a switch to cabergoline may be attempted [3,14].

Estradiol is the most potent estrogen produced in the body and is synthesized from testosterone or estrone. Aromatase, also known as estrogen synthetase, is the key enzyme in estrogen biosynthesis. Studies have shown that aromatase activity is not limited to the gonads and placenta, but the enzyme also acts in the brain, fat tissue, vascular tissue, bone, hair, and muscle [15].

Aromatase inhibitors, such as letrozole and anastrozole, act by blocking the aromatization of androgen to estrogen, resulting in decreased estrogen concentrations, and are thus traditionally used as an adjuvant therapy in post-menopausal women with estrogen-receptor-positive breast cancer [16].

The aim of this systematic review was to evaluate the effect of aromatase inhibitors on male prolactinoma with dopamine-agonist-resistant or persistent hypogonadism following treatment.

## 2. Materials and Methods

**Figure 1:**
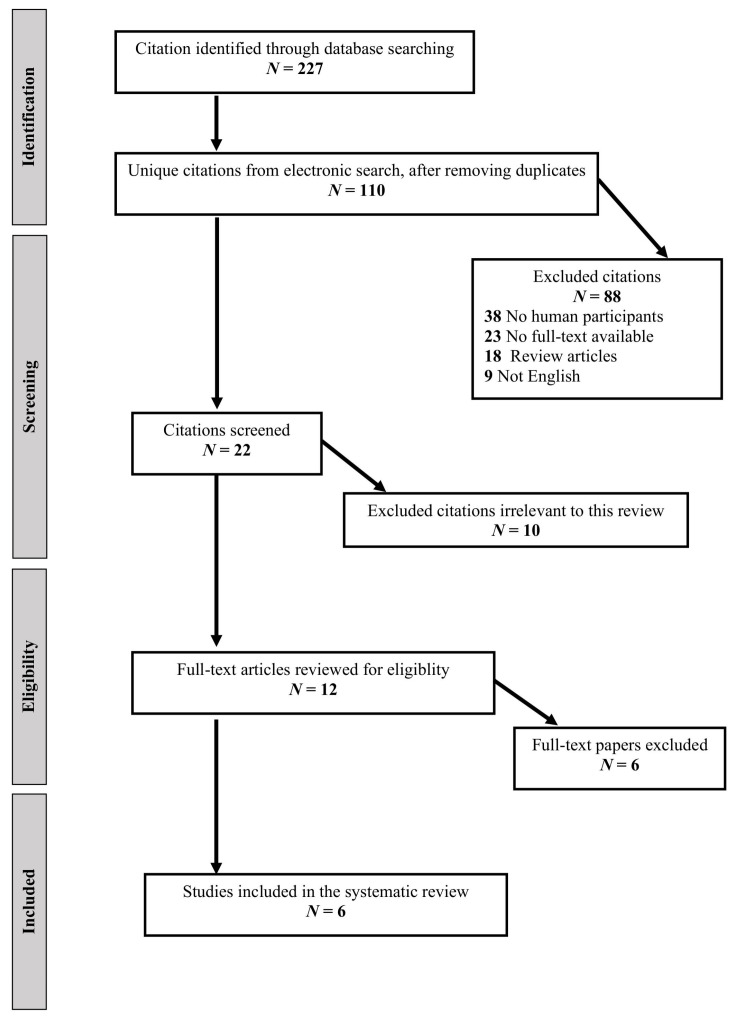
Systematic review flow diagram.

We performed a systematic review according to the Preferred Reporting Items for Systematic Reviews and Meta-Analyses (PRISMA) statement of studies (Moher et al., 2009). The literature databases were searched from their inceptions up to 1 December 2022 for relevant peer-reviewed articles written in English; we used the Medline Ovid, Medline (PubMed), Web of Science, and Google Scholar databases. The terms included in the search string were combined with Boolean operators as follows: ((“Prolactin” OR “Hyperprolactinemia” OR “Prolactinoma”) AND (“Aromatase Inhibitor”)). We supplemented the electronic search by cross-referencing the included papers, relevant sections of clinical practice guidelines, and relevant systematic and narrative reviews. Two authors (A.A. and Y.R.) conducted the search; articles were first assessed by title, then by abstract, and finally by the full text. Original studies in adults ≥18 years of age with an observational design (case series, case reports, cross-sectional, case–control, and cohort) reporting data on the clinical and biochemical characteristics of aromatase inhibitors for men with prolactinomas were included. Studies in children and adolescents, non-English articles, articles without pertinent data, and articles without full-text availability were excluded (Figure 1). The trial characteristics extracted included the sample size and number of patients treated with aromatase inhibitors, the specific medication and dose used, and biochemical data, including serum prolactin, testosterone, and estrogen levels. The manuscript was originally designed as a narrative review but, following the review process, it was redesigned as a systematic review. As we had already conducted the literature review beforehand, our review did not fulfill the requirement to register the protocol before the literature search and the review and the protocol were not registered.

## 3. Results

As detailed in the flow diagram (Figure 1), we retrieved a total of 227 citations from our electronic searches, ultimately yielding 110 unique citations after removing any duplicates. References from the manual search were all included in the electronic database searches. We reviewed 10 full-text papers for eligibility, and 6 studies published between 2002 and 2021 met the inclusion criteria and were included in the systematic review [17,18,19,20,21,22]. A summary of the trials included in the systematic review is shown in Table 1. Of the included studies, five were case reports, each reporting on a single case [17,18,19,20,22], and one study was a case series reporting on four patients [21]. Anastrozole was the aromatase inhibitor used in four of the studies [18,19,21,22], while letrozole was used in two cases [17,20] (Table 1).

In 2002, Gillam and colleagues reported on a case of a 34-year-old male patient with a macroprolactinoma and low testosterone levels; the patient responded to cabergoline treatment with significant reduction in prolactin levels, from 10,362 µf/L to 61 µg/L. However, as symptoms of hypogonadism persisted, testosterone was added, followed by an increase in prolactin levels, which came back down following the discontinuation of testosterone. A combination of cabergoline with anastrozole (1 mg daily) and a testosterone treatment resulted in improved clinical symptoms without increasing prolactin levels. Interestingly, at attempt to discontinue the anastrozole to determine the effect of anastrozole on prolactin levels resulted in an immediate spike in the prolactin levels, with a subsequent decline after re-introducing the aromatase inhibitor. The authors concluded that the addition of an aromatase inhibitor allowed for the continued use of testosterone as it prevented the testosterone-induced prolactin increase and potential tumor enlargement. Furthermore, the combination of an aromatase inhibitor with a dopamine agonist resulted in prolactin levels that were lower than those recorded at any other time point, even when the patient was on a weekly cabergoline dose of 21 mg; this finding supports the theory that reducing estrogen levels with an aromatase inhibitor improves sensitivity to dopamine agonists [22].

In 2010, Heidari and colleagues reported on a 36-year-old infertile man with persistent hypogonadism who received either bromocriptine or cabergoline treatment. The introduction of testosterone replacement or human chorionic gonadotropin (hCG) resulted in increased prolactin levels, which declined following treatment discontinuation. The patient was treated with cabergoline, which was increased up to 5 mg weekly, before beginning testosterone replacement therapy. As prolactin levels increased after adding the testosterone therapy, the cabergoline dosage was further increased to 7.5 mg weekly, before switching to bromocriptine due to cost issues. When a combination of bromocriptine, hCG, and letrozole (2.5 mg daily) was introduced to achieve fertility, prolactin levels decreased by up to 75%, to near-normal levels, in association with tumor shrinkage, the recovery of serum testosterone and sexual function, and improved sperm count and fertility. Once more, the authors speculated that inhibiting the aromatization of testosterone to estradiol reduced estrogen levels, thus decreasing estrogen-stimulated prolactin release [20].

Lima and colleagues reported on a 29-year-old man with hyperprolactinemia exacerbated by testosterone replacement therapy. The patient was diagnosed with a giant prolactinoma and achieved normal serum prolactin levels with cabergoline. However, as symptoms of hypogonadism persisted, testosterone replacement therapy was initiated, followed by a more than six-fold increase in prolactin levels. Adding letrozole (2.5 mg daily) treatment was associated with a significant reduction in prolactin levels [17].

Burman and Link reported on a case of anastrozole-induced rapid normalization of prolactin in a man with a giant prolactinoma. The 34-year-old man was diagnosed with a giant prolactinoma that dislocated the optic chiasm and the left optic nerve; treatment with cabergoline resulted in normalization of his visual fields and a gradual decline in serum prolactin levels. However, as testosterone levels remained low at twelve months, treatment with testosterone replacement therapy was initiated, resulting in a three-fold increase in serum prolactin and estradiol. Introducing anastrozole rapidly normalized serum prolactin levels [19].

Ozturk and colleagues reported on a case of a 28-year-old man with macroprolactinoma and hypogonadotropic hypogonadism. As the initial diagnosis was a nonfunctional adenoma with a tumor compressing the optic chiasm, causing bitemporal hemi-anopsia, the patient underwent transsphenoidal surgery. Histological analysis revealed a pituitary adenoma with positive staining for prolactin. Following the surgical operation and subsequent reevaluation, it was concluded the tumor was a prolactinoma and treatment with cabergoline was initiated, leading to a marked decline in prolactin levels. However, as testosterone levels remained low, testosterone replacement therapy was started; this was followed by an abrupt increase in prolactin levels, with no response to gradual increments of the cabergoline dosage. Imaging showed no evidence of tumor enlargement. Prolactin levels decreased significantly following the discontinuation of the testosterone replacement therapy. As treatment with testosterone was the cause of the prolactin increase, it was decided to restart testosterone replacement therapy with 1 mg of anastrozole daily, along with cabergoline. This combination achieved normal prolactin and testosterone levels [18].

In 2021, Ceccato and colleagues described four male patients with cabergoline-resistant prolactinoma who were treated with anastrozole (1 mg daily) combined with the maximum tolerated dose of cabergoline. This combination resulted in a decline in prolactin levels and decreased tumor sizes in all patients. At 1 year, the mean decline in prolactin secretion was 70% (ranging from −44% to −97%), and one patient achieved normal prolactin levels. The mean tumor volume shrinkage was 47% (ranging from −25% to −69%) [21].

## 4. Discussion

This systematic review supports the use of a combination of a dopamine agonist with an aromatase inhibitor for a subgroup of male patients with prolactinoma, as the addition of anastrozole or letrozole improved the control of prolactin levels and may lead to shrinkage in tumor sizes. Some reports suggest that treatment with testosterone may result in a decrease in the efficacy of dopamine agonists [22,23]. This decrease in dopamine agonist sensitivity following the introduction of testosterone therapy is likely secondary to the aromatization of testosterone to estrogen, which can stimulate the proliferation and hyperplasia of lactotroph cells in the pituitary, inducing resistance to dopamine agonists [24]. Aromatase inhibitors are primarily valuable for patients with dopamine-agonist-resistant prolactinoma, or when hypogonadism persists while using high-dose dopamine agonists.

According to the Endocrine society guidelines, the treatment response of a prolactinoma is defined as the normalization of prolactin levels on maximally tolerated doses of dopamine agonists and a reduction of 50% in the size of the prolactinoma [3]. However, when there is a lack of response, it could be due to cabergoline-resistant tumors resulting from a decreased number of dopamine [2] receptors, a CSF leak, or intolerance to or poor compliance with cabergoline. In addition, it is suggested that some patients with cabergoline resistance have higher prolactin levels at diagnosis, and require longer periods for normalization [25].

While medical therapy is highly effective for most prolactin-secreting pituitary tumors, some cases are resistant to dopamine agonist therapy and are accompanied by hypogonadism persistence, and another treatment modality is required. There is no universal consensus on the definition of dopamine agonist resistance. Several criteria regarding the hormonal response have been suggested, including failure to normalize prolactin levels, failure to reduce prolactin sufficiently to achieve ovulation, or failure to reduce prolactin by ≥50% [26].

The prevalence of dopamine agonist resistance is 20–30% for bromocriptine and approximately 10% for cabergoline. Approximately 80% of patients who have bromocriptine-resistant tumors may achieve prolactin normalization using cabergoline, and most patients with cabergoline-resistant tumors can be expected to respond to larger doses [26,27,28]. A study by Delgrange et al. showed that resistance to the prolactin-lowering effect of cabergoline is often partial and can be overcome in 75% of cases by increasing the weekly dose up to 3.5 g; little additional advantage was seen above a threshold of 3.5 g/week [28]. Ono et al. found that the maximum dose of cabergoline used successfully was 11 mg/week [27].

Most patients with prolactinoma are expected to show a reduction in tumor size with a substantial reduction in prolactin levels; moreover, in most cases, a patient who has minimal or no PRL reduction will also have no reduction in tumor size. However, there can be discordance between these two outcomes. In such tumors, additional treatment modalities may be required, including neurosurgery, radiotherapy, and/or temozolomide [3,29].

The surgical removal of a prolactinoma may result in the resolution of hyperprolactinemia, especially with microadenomas that are particularly well suited to surgery. However, recurrence following surgical resection may occur postoperatively. In patients with large tumors, complete resection is difficult to achieve [30].

Radiation therapy, either fractionated external beam radiotherapy (EBRT), known as conventional radiotherapy, or stereotactic radiosurgery, may be effective for tumor control or shrinkage in patients for whom surgical intervention and dopamine agonist treatment fail, or for patients with an aggressive or malignant prolactinoma. However, the maximal effect may take several years to achieve, hypopituitarism may occur in a significant number of patients, and, albeit rarely, cranial nerve injury may develop or a second tumor may form [31].

Temozolomide is an oral alkylating agent with a response rate for aggressive or malignant prolactinomas of about 50%. Even if pituitary tumors initially respond to temozolomide treatment, they often develop a resistance to it, so a second course of temozolomide treatment after regrowth is almost always ineffective [29]. 

Hypogonadism in hyperprolactinemia may be reversible due to the inhibitory effect of prolactin on the gonadotrophic hormones from the prolactin, or irreversible, secondary to the destruction of gonadotrophic cells. The recovery of male hypogonadism following the successful treatment of prolactinoma was reported in only 10 of 26 men with hypogonadism, with a mean time to recovery of 8.8 months, indicating that fewer than 40% of the patients exhibited a recovery of the hypogonadal axis after the normalization of prolactin levels [32]. In our recent study of 58 men with macro-prolactinoma, 79% of male patients who were followed for 5.6 years achieved eugonadism, while 21% had persistence of hypogonadism. Our data show that low baseline testosterone levels, visual field defects, and pituitary hormone deficiency are predictors of the persistence of hypogonadism within the first year following prolactin normalization, a finding that may aid in identifying patients who are suitable for testosterone replacement therapy [33].

Hypogonadism in men is associated with an increased risk of developing metabolic syndrome, and testosterone replacement may improve several metabolic parameters, including the body mass index, waist circumference, lipid profile, and glucose metabolism [34].

While testosterone replacement therapy is an accepted therapy for hypogonadism in men with prolactinomas, it may interfere with the biochemical and structural responses to dopamine agonists, and may even stimulate tumor growth and secretion. Prior and colleagues were the first to report testosterone-induced exacerbation in a man with prolactinoma, describing a 37-year-old man with a macroprolactinoma that was initially treated with bromocriptine, resulting in a significant decrease in serum prolactin. Due to absent libido and impotence, testosterone enanthate was administered after 12 weeks, and was associated with a marked increase in prolactin levels and an increase in tumor size. After increasing the dose of bromocriptine, testosterone was administered once more, resulting in the doubling of serum prolactin levels. Adding the less aromatizable anabolic steroid stanazolol was associated with a significant decrease in serum prolactin levels, but the treatment was stopped after two weeks due to adverse effects [35]. A study by Sodi and colleagues supported the hypothesis that the aromatization of testosterone to estradiol results in increased prolactin secretion, reporting that testosterone replacement may increase prolactin secretion and that the hyperprolactinemia in these cases seems to be resistant to treatment with dopamine agonists. The authors suggested the use of non-aromatizable androgens, or else combining testosterone replacement treatment with an aromatase inhibitor [36].

Prolactin-secreting lactotroph cells residing in the pituitary gland express an estrogenic receptor, and estrogen stimulation of the lactotroph cells results in an increase in prolactin synthesis. Carretero and colleagues reported that aromatase is expressed in pituitary adenomas in rats and is most evident in prolactin-secreting tumors, suggesting that an abnormally high conversion of testosterone to estradiol in pituitary cells may contribute to the development of prolactin-secreting pituitary adenomas [37]. Akinchi and colleagues evaluated human pituitary tissue samples of patients who had adenomectomies after a diagnosis of prolactinoma, as well as human pituitary tissue from autopsies; they found higher-than-normal aromatase expression in the pituitary tissues of patients with prolactinoma. The expression was higher in men with invasive adenomas compared to males without invasive tumors. The authors found no association between aromatase cytochrome P450 enzyme intensity and resistance and remission in patients with prolactinoma [38]. Similarly, Su and colleagues reported on the increased expression of the aromatase cytochrome P450 enzyme in invasive prolactinoma tissue in post-menopausal women, compared with non-invasive prolactinoma and the expression of estrogen receptor-β was found to be associated with dopamine agonist resistance, suggesting that the aromatization of testosterone to estrogen may play a role in the aggressiveness of prolactinomas [39]. Garcia-Barrado and colleagues reported that aromatase synthesized in the pituitary gland can produce estradiol locally and is overexpressed in prolactinomas. These findings support the potential role of aromatase inhibitors in prolactinomas, especially dopamine-agonist-resistant tumors [40].

Anastrozole and letrozole are nonsteroidal enzyme inhibitors, which inhibit enzyme activity by binding with the heme iron of the enzyme. While anastrozole and letrozole are potent and almost completely inhibit aromatase, neither medication suppresse plasma estradiol completely due to the high plasma concentration in testosterone, a precursor for estradiol synthesis in men. This is a potential explanation for the lack of significant side effects of aromatase inhibitors in men [15].

The negative effect of aromatase inhibitors on bone metabolism, with increased bone resorption and decreased bone mineral density, is the primary concern regarding the use of anastrozole and letrozole. Additional potential adverse effects may include loss of libido, especially with letrozole. The excess androgens associated with both medications may be associated with decreased HDL cholesterol levels and increased hemoglobin levels.

An increase in liver enzyme has been reported in approximately 10% of treated patients. Additional potential adverse effects include a rash, dry mouth, fatigue or weakness, nausea, changes in bowel movements, joint and tendon pain, mood changes, and sleep disorders [15,41].

The available data in the literature regarding aromatase inhibitors for prolactinoma are limited to male patients and, at the present time, the use of aromatase inhibitors for prolactinoma treatment is considered off-label.

However, aromatase inhibitors are safe for use in post-menopausal women, as they have been used to treat estrogen-receptor-positive breast cancer and have a well-documented safety profile.

Notably, in male patients with prolactinomas and persistent hypogonadism under treatment with dopamine agonists, the use of clomiphene citrate, a selective estrogen receptor modulator that increases gonadotropin secretion via hypothalamic-pituitary action, has been shown to improve testosterone levels, erectile function, and sperm motility. The effects of selective estrogen receptor modulators on testosterone levels were not secondary to decreasing prolactin levels, but arose due to increasing gonadotropin levels and unchanged prolactin levels, suggesting that clomiphene may be valuable for patients who wish to have children [42].

As most of the effects of estrogen are mediated though alpha and beta estrogen receptors, the use of fulvestrant, an ESR1 antagonist used for the treatment of post-menopausal women with hormone-sensitive breast cancer, could inhibit the growth of pituitary adenomas through inducing apoptosis, and may be valuable in some cases [43].

## 5. Conclusions

There are preliminary data to suggest that aromatase inhibitors may play a role in the treatment of men with prolactinoma. The inhibition of the aromatization of testosterone to estradiol leads to lower estrogen levels with decreased estrogen-stimulated prolactin release. Aromatase inhibitors may be most valuable for patients with dopamine-agonist-resistant prolactinoma, or when hypogonadism persists while using high-dose dopamine agonists.

## Figures and Tables

**Table 1:** Systematic review: summary of available data. Previously conducted case reports and case-series on the use of aromatase inhibitors for male patients with prolactinoma.

Study	Patients	Age	Adenoma Size	Baseline Prolactin Levels	Maximal Cabergoline Dose	Nadir of Prolactin Levels During Cabergoline	Timing of Testosterone Replacement Therapy	Maximal Prolactin on Testosterone	Aromatase Inhibitor Treatment	Minimal Prolactin Levels on Aromatase Inhibitor	Tumor Mass
Gillam et al., 2002 [22]	1	34 y/o	4.5 × 3.5 × 4.5 cm	10,362 µg/L (2.7–12.2)	21 mg weekly	71 µg/L (2.7–12.2)	At 16 months for 1 month; at 22 months.	295 µg/L (2.7–12.2)	Anastrozole, 1 mg daily	36 µg/L (2.7–12.2)	No data
Heidari et al., 2010 [20]	1	36 y/o	2.0 × 1.8 × 1.5 cm	420 ng/mL	5 mg weekly	23 ng/mL	At 6 months	96 ng/mL. d/t desire for fertility, hCG was initiated and PRL increased to 221 ng/mL	Letrozole, 2.5 mg daily	29 ng/mL	Shrinkage
Lima et al., 2013 [17]	1	29 y/o	3.0 × 4.5 × 3.5 cm	1218 ng/mL (<10 ng/mL)	1.5 mg weekly	<10 ng/mL	At 24 months	60 ng/mL (<10 ng/mL)	Letrozole, 2.5 mg daily	24 ng/mL (<10 ng/mL)	Shrinkage
Burman et al., 2016 [19]	1	34 y/o	Giant prolactinoma	360,430 mU/L (<400 mU/L)	5 mg weekly	556 mU/L (<400 mU/L)	At 12 months	1477 mU/L (<400 mU/L)	Anastrozole	<400 mU/L (<400 mU/L)	Shrinkage
Ozturk et al., 2017 [18]	1	28 y/o	3.6 × 3.5 × 2.3 cm	323 ng/mL	1 mg weekly	80 ng/mL	At 2 months	470 ng/mL	Anastrozole, 1 mg daily	18.8 ng/mL	No enlargment
Ceccato et al., 2021 [21]	4	26 y/o	3.3 × 2.3 × 3.5	14,000 µg/L (<15 µg/L)	4.5 mg weekly	1920 µg/L (<15 µg/L)	NA	NA	Anastrozole, 1 mg daily	50 µg/L (<15 µg/L)	Shrinkage
		38 y/o	5.2 × 4.8 × 5.0	33,000 µg/L (<15 µg/L)	4.5 mg weekly	270 µg/L (<15 µg/L)	NA	NA	Anastrozole, 1 mg daily	23 µg/L (<15 µg/L)	Shrinkage
		29 y/o	1.7 × 1.4 × 1.5	1460 µg/L (<15 µg/L)	3 mg weekly	35 µg/L (<15 µg/L)	NA	NA	Anastrozole, 1 mg daily	18 µg/L (<15 µg/L)	Shrinkage
		19 y/o	1.3 × 1.5 × 1.0	850 µg/L (<15 µg/L)	3.5 mg weekly	26 µg/L (<15 µg/L)	NA	NA	Anastrozole, 1 mg daily	14 µg/L (<15 µg/L)	Shrinkage

y/o—years old; NA—not available; PRL—prolactin; hCG—human chorionic gonadotropin.

## Data Availability

All data are provided within the article.
